# Too Much of a Good Thing: Random Practice Scheduling and Self-Control of Feedback Lead to Unique but Not Additive Learning Benefits

**DOI:** 10.3389/fpsyg.2012.00503

**Published:** 2012-12-10

**Authors:** Asif Ali, Bradley Fawver, Jingu Kim, Jeffrey Fairbrother, Christopher M. Janelle

**Affiliations:** ^1^Kyungpook National UniversityDaegu, South Korea; ^2^University of FloridaGainesville, FL, USA; ^3^University of TennesseeKnoxville, TN, USA

**Keywords:** accuracy, anticipation timing, challenge, consistency, variability

## Abstract

We examined the impact of self-controlled knowledge of results on the acquisition, retention, and transfer of anticipation timing skill as a function of random and blocked practice schedules. Forty-eight undergraduate students were divided into experimental groups that practiced under varying combinations of random or blocked as well as self-controlled or yoked practice conditions. Anticipation timing performance (5, 13, and 21 mph) was recorded during acquisition and during a short term no-feedback retention test. A transfer test, administered 24 h after the retention test, consisted of two novel anticipation timing speeds (9, 17 mph). Absolute error (AE) and variable error (VE) of timing served as the dependent measures. All participants improved their accuracy and consistency across acquisition blocks; however, those who practiced under blocked rather than random conditions had greater accuracy (lower AE) regardless of feedback delivery. During retention and transfer, those who practiced under random conditions showed greater consistency (lower VE) compared to their blocked counterparts. Finally, participants who controlled their feedback schedule were more accurate (lower AE) and less variable (lower VE) during transfer compared to yoked participants, regardless of practice scheduling. Our findings indicate that practicing under a random schedule improves retention and transfer consistency, while self-control of feedback is advantageous to both the accuracy and consistency with which anticipation timing skill transfers to novel task demands. The combination of these learning manipulations, however, does not improve skill retention or transfer above and beyond their orthogonal effects.

## Introduction

In many learning settings, augmented feedback is critical for skill acquisition and refinement (Salmoni et al., 1984; Adams, 1987). Knowledge of results (KR) is a form of augmented feedback that provides outcome related information concerning how well a task was performed. A large body of literature provides compelling evidence that KR enhances motor learning in a variety of ways (e.g., McNevin et al., 1994; Guadagnoli et al., 1996; Shea and Wulf, 1999; Fredenburg et al., 2001; Guadagnoli and Kohl, 2001; van Vliet and Wulf, 2006). Traditionally, KR has been provided to the learner according to a set schedule determined beforehand by the experimenter, but researchers have become increasingly interested in feedback manipulations that shift at least some control of feedback delivery to the learners.

### Self-controlled feedback

Self-controlled feedback (SCFB) schedules allow learners to decide when they will receive feedback, which gives them control over when feedback is administered (e.g., after attempts they perceive to be relatively “good” or “poor,” or when they deem it most relevant for error correction), the overall frequency of feedback (i.e., how many trials are paired with feedback), and how to change the frequency as practice progresses (e.g., to create a faded schedule that reduces the relative and absolute frequency of feedback across the practice session).

Self-controlled feedback has been established as an effective means of facilitating motor learning for a variety of tasks such as object tossing (Janelle et al., 1995; Chiviacowsky et al., 2008a,b), basketball set shot (Aiken et al., 2012), and key pressing (Chen et al., 2002; Chiviacowsky and Wulf, 2002, 2005; Patterson and Carter, 2010; Hansen et al., 2011). Purported mechanisms for SCFB effects include increased motivation (Janelle et al., 1995, 1997; Chiviacowsky and Wulf, 2002; Chiviacowsky et al., 2008a; Wulf et al., 2010), subjective error estimation (Chiviacowsky and Wulf, 2005), and enhanced information processing (Janelle et al., 1995, 1997; Wulf et al., 2005).

Manipulations of self-control (in addition to feedback) have been shown to benefit learning for a variety of other types of instructional support, including physical guidance (Wulf and Toole, 1999), total amount of practice (Post et al., 2011), video demonstration (Bund and Wiemeyer, 2004; Wulf et al., 2005), and the practice schedule itself (Bund and Wiemeyer, 2004; Keetch and Lee, 2007; Bastos et al., 2010; Hodges et al., 2011; Wu and Magill, 2011). The latter set of findings is intriguing because *the established benefits of SCFB have thus far been limited to single task learning situations* (e.g., Janelle et al., 1995, 1997; Chiviacowsky and Wulf, 2002, 2005; Wulf et al., 2005). It therefore remains unknown how SCFB might interact with traditional practice schedule manipulations such as those seen in comparisons of blocked and random practice effects (Shea and Morgan, 1979). It has been argued that both feedback and practice schedule manipulations can alter the functional demand of a learning situation and ultimately influence motor learning (Guadagnoli and Lee, 2004). Accordingly, our examination of the effects of SCFB on learning in blocked and random practice schedules represents a test of the generalizability of SC effects to different practice structures known to also influence motor learning.

### Blocked and random practice schedules

Shea and Morgan (1979) reported the first evidence that motor learning would be facilitated by practicing multiple tasks according to a random schedule. Since then, a large body of evidence has documented the performance and learning effects of blocked and random practice schedules when acquiring a variety of different skills (Magill and Hall, 1990). In a blocked practice schedule, participants practice a single task over consecutive trials before moving to other tasks (e.g., AAA…, BBB…, CCC…). In contrast, a random practice schedule presents tasks in an unsystematic fashion (e.g., ABA…, CAB…, BCC…). Typically, random practice schedules are created with the stipulations that no task will be performed more than two times in immediate succession, and that each trial block will present an equal number of trials on each task. Blocked practice schedules typically facilitate immediate performance during skill acquisition when compared to random schedules (Simon and Bjork, 2001; Shebilske et al., 2006; Choi et al., 2008). During retention and transfer tests, however, random practice schedules have frequently been shown to facilitate learning compared to blocked schedules (e.g., Lee and Magill, 1983; Fairbrother et al., 2002, 2007; Lee and Simon, 2004; Brydges et al., 2007; Porter and Magill, 2010).

Blocked practice schedules are thought to produce relatively low levels of *contextual interference* during skill acquisition, so immediate performance is facilitated relative to higher contextual interference conditions created with practice schedules that present tasks quasi-randomly or even in a serial order (see Lee and Magill, 1983). Prominent explanations of practice schedule effects rely on the idea that higher levels of contextual interference introduced during practice result in deeper information processing that ultimately facilitates learning compared to conditions that do not face such a challenge (Lee and Magill, 1983; Shea and Zimny, 1983, 1989). Despite substantial evidence that random practice facilitates learning compared to blocked practice, there is also evidence indicating that random practice is not always advantageous (Del Rey et al., 1987; Aloupis et al., 1996; Guadagnoli et al., 1999).

Practice schedules that cause *high levels of contextual interference may present too great of a challenge for learners*. For example, Wrisberg and Mead (1983) found a learning advantage for blocked practice (cf. to random) for young children learning an anticipation timing task. Similarly, Landin and Hebert (1997) found that a schedule designed to introduce an intermediate level of contextual interference facilitated the learning of a basketball set shot by participants with a moderate amount of experience. Although research suggests that participants may choose their practice schedule in a manner that increases CI (Wu and Magill, 2011). Wulf and Shea (2002) argued that practice structures that increase the challenge in a learning setting will not facilitate learning of complex tasks that impose a high processing load because their combination will simply be too challenging for learners. For relatively difficult tasks learning should be facilitated by a blocked practice schedule that prevents the challenge from becoming too great.

As a general rule, reduced relative frequency of KR has been shown to degrade immediate performance but to facilitate learning (Salmoni et al., 1984). Presumably, the provision of self-control over the administration of feedback should allow learners to fine-tune the challenge they face when presented with either a blocked or random practice schedule. For example, learners might choose to request a relatively high frequency of feedback to offset the challenge imposed by a random practice schedule. Similarly, learners might not need or prefer feedback as frequently when learning in blocked schedule.

Evidence indicates that SCFB participants strategically tailor feedback administration during practice. For example, some studies have reported relatively low KR request frequencies (e.g., Janelle et al., 1997; Chiviacowsky and Wulf, 2002; Patterson and Carter, 2010), while others have shown that participants create a faded-feedback schedule by reducing requests as practice progresses (e.g., Janelle et al., 1997; Fairbrother et al., 2012). Chiviacowsky and Wulf (2002, 2007) suggested that the benefits of SCFB are related to participants’ capabilities to self-evaluate their performance and tailor feedback requests to match their learning needs or preferences. This argument raises the question of whether or not different practice schedules might influence a participant’s capability to use SCFB according to their individual needs. On the one hand, it seems plausible that the effects of SCFB might operate independently of those produced by practice schedule manipulations. In other words, the previous demonstrations of the generalizability of SC manipulations suggest that a benefit should be seen regardless of the challenge presented by the practice schedule. On the other hand, it’s also possible that the challenge imposed by random practice might change behavior in ways that will influence SCFB effects.

The purpose of this investigation was, therefore, to examine the independent and combined effects of practice schedule (blocked or random) and feedback (self or yoked manipulations) on the performance and learning of a simple motor skill. Two primary hypotheses were offered for the practice scheduling and feedback manipulation main effects, respectively. Consistent with prior work (e.g., Fairbrother and Brueckner, 2008), we expected that switching between anticipation timing options (variable speeds) in a random practice arrangement would result in degraded performance but enhanced learning (better retention and transfer) of the anticipation timing task compared to conditions that repeated a single timing variation. Likewise, the SCFB advantages reported in prior work (Janelle et al., 1997; Chiviacowsky et al., 2008a,b; Fairbrother et al., 2012) were expected to be evidenced herein by better retention and transfer performance.

Tentative assertions were also made concerning the expected interactions of the two practice manipulations. Considering both participant skill and task difficulty, past research has indicated that anticipation timing tasks, albeit novel, present rather low task difficulty. As such, we expected that for this task, which required low levels of KR for both blocked and random practice conditions, the effects of practice schedule would interact with and be augmented by the benefits of SCFB. More specifically, while performance was expected to suffer (relative to blocked conditions) during acquisition, we expected that those who learned under random practice conditions but who could control feedback frequency would demonstrate better retention and transfer to novel anticipation timing speeds.

## Materials and Methods

### Participants

Forty-eight undergraduate students (24 women) with a mean age of 22.62 years (SD = 2.61) participated in this experiment for extra course credit. Male and female participants were placed in equal numbers in each experimental group. All participants were randomly assigned evenly to a practice schedule (Random vs. Blocked) and then randomly assigned to a feedback schedule (Self-controlled vs. Yoked), which yielded four experimental groups: (1) Self-Controlled KR-Random (SC-R), (2) Self-Controlled KR-Blocked (SC-B), (3) Yoked KR-Random (YK-R), and (4) Yoked KR-Blocked (YK-B). Participants had no prior experience with the task used in this study and were naïve to the purpose of the experiment. All participants signed informed consent forms prior to the experiment approved by the Institutional Review Board at Kyungpook National University.

### Apparatus and task

A Bassin Anticipation Timer (BAT: Model# 50575, Lafayette Instrumental Company, Lafayette, IN, USA) was used as the experimental apparatus. The apparatus runway was 1.44 m long. Participants sat 3 m in front of the target light of the BAT and were asked to press the response button with the thumb of their dominant hand at the exact time when the sequential illumination of approaching lights reached the target light. The stream of lights ran from left to right toward the target light located on the right end of the timer. A yellow colored warning light on the left end of the timer indicated the initiation of the trial. Three different speeds of the runway were used: 5 mph (slow), 13 mph (moderate), and 21 mph (fast), for acquisition and the no-feedback retention test (10 min later). A transfer test was administered 24 h after the retention test, at two novel speeds (9 and 17 mph).

### Procedure

Upon arriving in the laboratory, participants were provided with instructions concerning the task and the feedback provision procedure. Three practice trials, including one trial of each slow, moderate, and fast target speed, were administered to familiarize participants with the apparatus and task. The experimenter instructed participants that the warning light would appear for 1.5 s before the start of the trial. They were further instructed that the experimenter would say “ready” before the initiation of each trial and then immediately press the start button of the timer. During acquisition, verbal KR comprising the direction and magnitude of errors was available following the trial performance for self-controlled participants whenever they requested it, and for yoked participants according to a schedule produced by their counterparts in the self-controlled condition. KR was provided in terms of early (−), zero (0) or late (+) error, in milliseconds.

During the acquisition phase, all participants completed 90 total trials of each of the three speeds (e.g., slow, moderate, and fast). Participants in SC-R and YK-R groups completed the 90 trials in three blocks of 30 trials using a random practice schedule. The participants in SC-B and YK-B group executed 30 trials of each of three speeds in three blocks, consisting of 90 trials in total, according to the blocked practice schedule. In the blocked condition, 30 trials of each of the three slow, moderate, and fast speeds were completed consecutively during the trial block. The retention test consisted of 30 trials, of three speeds in a serial order (i.e., slow, moderate, fast, slow, moderate, fast), to minimize learning effects during testing. The inter-trial interval was 5 s, during which data was recorded and feedback was provided during the acquisition trials. To assess stability and long-term learning, a transfer test (20 trials) was conducted approximately 24 h after the experiment; consisting of 10 trials of each of two novel speeds, in an alternating sequence (i.e., 9, 17, 9, 17 mph). A rest interval of 1 min was provided for all groups between each trial block. All participants performed the retention and transfer test without KR.

### Data reduction

Dependent measures included absolute error (AE) and variable error (VE). AE was calculated by taking the absolute value of each error score across each of the three trial blocks, resulting in an unbiased measure of total error. Additionally, AE was less sensitive to potential outliers compared to other measures of total error, such as root-mean-square error. VE was calculated by taking the standard deviation between the total reaction times and the mean reaction time, thereby providing a measure of consistency across trials. Prior to all subsequent analysis, data was collapsed across speeds within each of the acquisition, retention, and transfer portions of the experiment.

### Statistical analysis

To determine performance differences during the acquisition phase, dependent measures were each analyzed in a separate Feedback (Self-controlled, Yoked) × Practice (Blocked, Random) × Trial Block (1, 2, 3) mixed model analysis of variance (ANOVA) with repeated measures on the last factor. For the retention and transfer phases, dependent measures were analyzed in separate Feedback (Self-controlled, Yoked) × Practice (Blocked, Random) ANOVAs, in which data were collapsed across trial blocks. Bonferroni adjusted *t*-tests and simple effects tests were implemented as follow ups for significant main effects and interactions, respectively. Greenhouse–Geisser degrees of freedom corrections were applied for ANOVAs where the sphericity assumption was violated. The critical level of significance was established at *p* < 0.05 for all statistical analyses.

## Results

On average, participants in the SC-R and SC-B groups asked for KR during 8 and 11% of trials respectively during acquisition. Breaking down KR requests separately during the three trials blocks revealed that the SC-R group requested feedback on 8.6%, 8.1%, and 6.7% of the total trials in blocks 1–3, respectively, and the SC-B group requested feedback on 15%, 10%, and 8.6% of the total trials in those blocks.

### Absolute error

**Figure 1:**
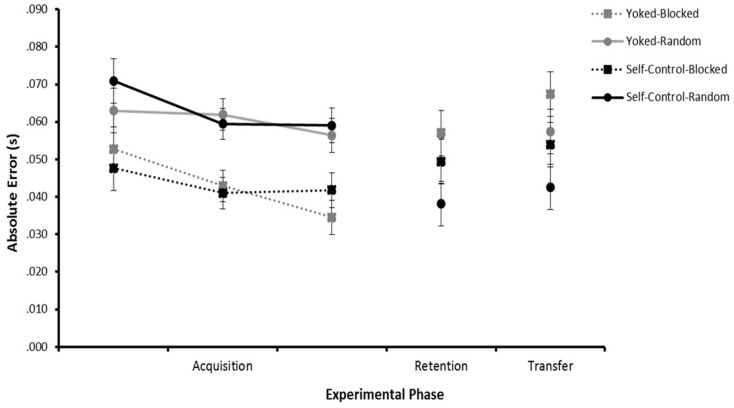
**Absolute error across experimental phases**. Acquisition (trial blocks 1–3) and Retention (combined trial blocks 4–5) data represent absolute error collapsed across speed (5, 13, 21 mph). Transfer (combined trial blocks 6–7) data represents absolute error collapsed across speed (9 and 17 mph).

Absolute Error scores for each trial represented an unbiased measure of total error (see Figure 1).

#### Acquisition

The analysis of AE during acquisition revealed a significant main effect for Practice, *F*(1, 44) = 23.657, *p* < 0.001, η^2^ = 0.350, and Trial Block, *F*(2, 88) = 7.671, *p* = 0.001, η^2^ = 0.148. Follow-up tests revealed that, although participants improved accuracy over trial blocks, participants who practiced under random conditions were less accurate compared to those who practiced under blocked conditions during the acquisition phase. The main effect for Feedback, *F*(1, 44) = 0.137, *p* > 0.05, η^2^ = 0.003, and the interaction of Feedback × Practice, *F*(1, 44) = 0.121, *p* > 0.05, η^2^ = 0.003, were not significant.

#### Retention

Analysis of AE during retention revealed no significant main effects for Practice, *F*(1, 44) = 2.809, *p* > 0.05, η^2^ = 0.060, Feedback, *F*(1, 44) = 2.929, *p* > 0.05, η^2^ = 0.062, or the interaction of Practice × Feedback, *F*(1, 44) = 0.096, *p* > 0.05, η^2^ = 0.002, main effects, along with all other interactions were not significant.

#### Transfer

**Figure 2:**
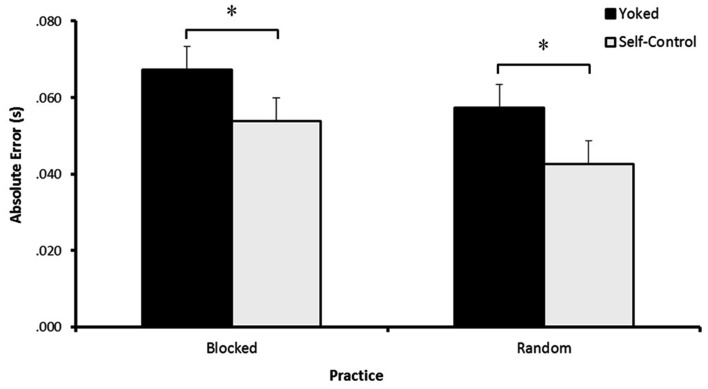
**Absolute error during transfer**. Participants who controlled their feedback schedule during practice (SC-R and SC-B) had improved accuracy, indicated by lower error scores, compared to yoked participants (YK-R and YK-B). * Indicates *p* < 0.05.

Analysis of AE during transfer revealed a significant main effect for Feedback, *F*(1, 44) = 5.601, *p* = 0.022, η^2^ = 0.113. Follow ups revealed that SCFB participants had less error compared to yoked participants (see Figure 2). The main effect of Practice, *F*(1, 44) = 3.157, *p* > 0.05, η^2^ = 0.067, and the interaction of Feedback × Practice, *F*(1, 44) = 0.012, *p* > 0.05, η^2^ < 0.001, were not significant.

### Variable error

**Figure 3:**
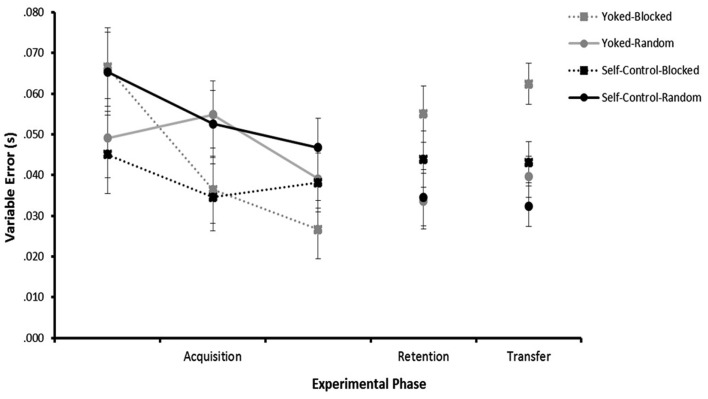
**Variable error across trial blocks**. Acquisition (trial blocks 1–3) and Retention (combined trial blocks 4–5) data represent variable error collapsed across speed (5, 13, 21 mph). Transfer (combined trial blocks 6–7) data represents variable error collapsed across speed (9 and 17 mph).

Variable Error scores represented the difference between all reaction times and the mean reaction time, indicating the level of variability of participants’ responses (see Figure 3). Lower scores correspond to increased consistency in the task.

#### Acquisition

Analysis of VE during acquisition revealed a significant main effect for Trial Block, *F*(2, 88) = 6.876, *p* = 0.002, η^2^ = 0.135. Follow-up tests revealed decreased variability from the first block to the third block. The main effects of Feedback, *F*(1, 44) = 0.076, *p* > 0.05, η^2^ = 0.002, Practice, *F*(1, 44) = 2.841, *p* > 0.05, η^2^ = 0.061, and the interaction of Feedback × Practice, *F*(1, 44) = 0.876, *p* > 0.05, η^2^ = 0.019, were not significant.

#### Retention

Analysis of VE during retention revealed a significant main effect for Practice, *F*(1, 44) = 5.009, *p* = 0.030, η^2^ = 0.102. Follow-up tests revealed less variability for participants who practiced under random conditions compared to the blocked conditions. The main effects for Feedback, *F*(1, 44) = 0.545, *p* > 0.05, η^2^ = 0.012, and the interaction of Feedback × Practice, *F*(1, 44) = 0.743, *p* > 0.05, η^2^ = 0.017, were not significant.

#### Transfer

**Figure 4:**
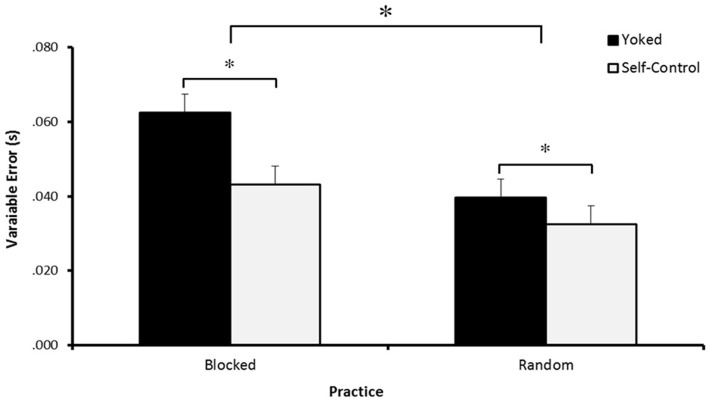
**Variable error during Transfer**. Participants who controlled their feedback schedule during practice (SC-R and SC-B) show reduced variability compared to those with a yoked feedback schedule (YK-R and YK-B). Additionally, participants who practiced under a random schedule (SC-R and YK-R) show reduced variability compared to those with a rigid practice schedule (SC-B and YK-B).* Indicates *p* < 0.05.

Analysis of VE during transfer revealed a significant main effect for Feedback. Additionally, participants who practiced under random conditions had decreased variability compared to those who practiced under blocked conditions (see Figure 4). The interaction of Feedback × Practice, *F*(1, 44) = 1.428, *p* > 0.05, η^2^ = 0.031, was not significant.

## Discussion

We sought to determine the impact of self-controlled KR on the learning and performance of an anticipation timing task practiced under random and blocked practice schedules. Results generally corroborate previous evidence that practicing under random conditions or SCFB conditions during skill acquisition offers advantages to retention and transfer of skilled learning. Specifically, practicing under random conditions improved timing consistency during retention and transfer, while control over feedback during skill acquisition improved performance accuracy and consistency during transfer. Although these practice manipulations are individually effective in improving skill learning, results indicate that they apparently provide no additive benefits when combined, at least when learning anticipation timing tasks such as the one of interest here. These results are further elaborated by considering the context specific CI and practice scheduling demands of the current investigation.

Participants in all four experimental conditions reduced errors and improved their accuracy in anticipation timing across trial blocks during acquisition. Those who practiced in a blocked arrangement, however, performed better during acquisition than those who practiced randomly, which is in line with previous work (Simon and Bjork, 2001; Shebilske et al., 2006; Choi et al., 2008). This result is not explained by the control of feedback, as the relative frequency of requests for feedback for blocked 11% and random conditions 8% were low and undifferentiated during acquisition. Additionally, these frequencies are consistent with studies that have traditionally used constant practice schedules in the SCFB literature.

Several factors likely explain improved performance during acquisition for those who practice under blocked conditions. First, participants in blocked conditions were expected to more accurately judge the speed of subsequent trials because consecutive trials were identical. As such, the inter-trial interval can be used to refine the template for similar responses, which resulted in better error estimation and immediate performance enhancement (Chiviacowsky and Wulf, 2005). Under random conditions, the number of task switches over trials is higher than blocked conditions, which typically increases error scores in early trials. Indeed, previous work has also identified poorer performance in switched conditions compared to repeated conditions during practice (Fairbrother and Brueckner, 2008; Sherwood, 2008). Moreover, Hsieh and Liu (2005) showed that task switching compromises stimulus identification and response selection. While long-term benefits may result, these processes are negatively impacted during early skill acquisition under random practice conditions.

Our findings corroborate previous work examining the effect of SCFB on motor learning during the practice of tasks in a constant order (e.g., Chiviacowsky and Wulf, 2002), but they do not support the expected advantage of SCFB over yoked feedback *during the acquisition period* for participants who practiced under a random schedule. In the current study, it is likely that learners may have found it difficult to know when to ask for KR, or how to apply it due to the randomness of the practice schedule. Although requests for feedback were similar, SC-R participants asked for feedback (8%) slightly less than the SC-B group (11%). Random practice schedules, by design, limit the ability to rely on a previous trial’s solution to aid in performance of an ensuing trial. As such, realizing that the information acquired may not pertain to the next trial, participants may have been reluctant or unable to develop a systematic strategy for feedback requests.

Paradoxically, the inability to use a systematic strategy for immediate changes may benefit long-term learning and flexibility in motor parameterization. Prior work has documented the systematic manner by which learners use SCFB for implementation of learning strategies. SCFB is effective particularly because participants choose specific “good” or “bad” trials in which to receive feedback, which maximizes performance improvements (Chiviacowsky and Wulf, 2002, 2005). In blocked or constant practice conditions, augmented information from a previous trial can be immediately applied to the ensuing trial. Such was not the case in the random arrangement, thereby increasing the difficulty of the task. That being the case, the information provided would be in reference to a previous trial; the performance of which in some way motivated that request for feedback. Participants most likely found it difficult to know when to ask the experimenter for KR or effectively use KR during random practice.

Apparently, for SCFB to be effective in reducing error and variability during acquisition, the learner must be able to appraise performance sufficiently enough to know when to ask for feedback. It is the uniqueness of trials chosen in self-controlled conditions that arguably allows the extraction of relevant performance information (Wulf et al., 2005). As opposed to choosing relevant trials on which to receive feedback, yoked control participants under random practice conditions would have received feedback in a comparatively arbitrary fashion. KR was arguably less interpretable and relevant for the yoked control group who received feedback sporadically, infrequently, inconsistently, and unrelated to their individual needs.

Previous work has shown a performance advantage during retention and transfer for those who have practiced using a random schedule (Shea and Morgan, 1979; Del Rey et al., 1983; Lee and Magill, 1983; Young et al., 1993; Lee and Simon, 2004; Keller et al., 2006; Brydges et al., 2007; Choi et al., 2008; Menayo et al., 2010; Porter and Magill, 2010; Travlos, 2010). The increase in CI due to random presentation of trials of varying speeds in the current study was not powerful enough to improve performance *accuracy* during retention or transfer; however, random practice participants were able to decrease timing *variability* compared to those that practiced in blocked conditions, regardless of whether or not feedback delivery was controlled by the learner. This result suggests that, for skills of nominal difficulty, random arrangements of trials during practice may improve participants’ ability to detect subtle changes in trial composition, which allows them to maintain a level of consistent responding, even during novel tasks.

Performance improvements during retention and transfer have also routinely been reported for those who control feedback during practice (Chen et al., 2002; Chiviacowsky and Wulf, 2002, 2005; Wulf et al., 2005; Patterson and Carter, 2010; Hansen et al., 2011). Predicted effects for those groups that controlled their feedback in both random and blocked groups were not substantiated during the no-feedback retention phase (although the group that requested their own feedback in a random practice schedule was the most accurate during this period). Apparently, memory consolidation over the 24 h delay between the retention and transfer tests was needed to realize the full benefits of the self-controlled random practice manipulations. Control over feedback conditions did result in performance improvements during transfer, with SC-R and SC-B participants holding an advantage over their yoked counterparts in both timing accuracy and consistency. Previous demonstrations of SC benefits have sometimes been isolated in transfer test results (e.g., Chiviacowsky and Wulf, 2002; Post et al., 2011), and it can be argued that relative to a retention test, transfer provides a more appropriate index of how learning generalizes to novel environments and conditions.

Based on the nominal task difficulty in the current experiment, we expected that the interaction between practice and feedback schedule manipulations would improve performance above and beyond the effect of each individual manipulation. Although visual inspection of the data suggests that the ability to self-control feedback when presented in a random practice arrangement leads to the best accuracy and consistency during retention and transfer, there is apparently no additive benefit of controlling feedback conditions during random practice in timing tasks of nominal difficulty. It is possible that participants during random practice were not able to make full use of the control over feedback conditions. Because of individual differences (e.g., skill differences in error detection ability) and the challenges imposed by the different practice schedules, participants within the same group may have requested feedback to either increase or decrease the difficulty of the task. Motives underlying the relative frequencies of requests could not be ascertained from the current study but should be the focus of future work in this area. Finally, it is possible that a task of this nature does not lend itself to the additive benefits of combined feedback and practice manipulations. It is well-known that this task is learnable, and participants can improve without feedback through discovery learning. Additive effects may be evident in other tasks which do not require timing anticipation or feature multiple degrees of freedom movements. Moreover, implementation of protocols in which learners self-control delivery of process-based (knowledge of performance) information random practice schedules may prove to be advantageous. While beyond the scope of this paper, the notion that SCFB allowed fine-tuning of the challenge presented by random practice is consistent with previous arguments that SC works by allowing participants to tailor practice to meet their needs and preferences (e.g., Chiviacowsky and Wulf, 2002). Future work that addresses how and why participants ask for feedback as a function of combined learning manipulations during acquisition would illuminate some of the intrinsic factors that drive performance improvements during retention and transfer.

In conclusion, our findings suggest that feedback and practice schedule manipulations provide differing benefits during retention and feedback phases of motor learning. Additionally, additive learning effects of feedback and practice schedule manipulations are not evident during anticipation timing tasks of nominal difficulty. Self-control over feedback in combination with a random practice schedule may indeed foster the development of a more malleable performance framework in other skills; however, further research is needed to examine any possible advantages during other tasks which use other movements or varying levels of difficulty. In addition, future investigations should test learning effects using different types and forms of feedback along with various practice schedules to advance our understanding of how to best facilitate the learning and retention of motor skills. Recent work (e.g., Hodges et al., 2011) has emphasized the need to consider such learning effects as a function of expertise. Such efforts should also be prioritized in future work to eventually be able to tailor optimal training recommendations to individuals across varying skill levels.

## Conflict of Interest Statement

The authors declare that the research was conducted in the absence of any commercial or financial relationships that could be construed as a potential conflict of interest.
